# Eculizumab in the Treatment of Gemcitabine-Induced Atypical Hemolytic Uremic Syndrome

**DOI:** 10.7759/cureus.35874

**Published:** 2023-03-07

**Authors:** Farhan Azad, Clive J Miranda, Al Amin, Ruhi Hadwani, Matthew Gravina

**Affiliations:** 1 Internal Medicine, University at Buffalo, Buffalo, USA; 2 Pathology, University at Buffalo, Buffalo, USA; 3 Hematology and Medical Oncology, University at Buffalo, Buffalo, USA

**Keywords:** eculizumab, uremic, klatskin, cisplatin, cholangiocarcinoma, gemcitabine

## Abstract

Gemcitabine-induced hemolytic uremic syndrome is an often-missed condition. We present a case outlining the successful management of a patient with metastatic cholangiocarcinoma treated with gemcitabine who subsequently developed hemolytic uremic syndrome. Early recognition and stopping gemcitabine are essential in this patient population. Complement inhibitors have been used, and our patient improved on eculizumab therapy.

## Introduction

Cholangiocarcinoma (CCA) is a biliary tumor comprising 15% of all primary liver tumors and 3% of gastrointestinal cancers. It is typically asymptomatic in the early stages and presents with jaundice, abdominal pain, nausea, and weight loss in advanced stages. Diagnosis requires histological confirmation, as non-invasive methods are not accurate [1]. The most relevant risk factors cited are primary sclerosing cholangitis, liver fluke infection, hepatitis C and B, duct cysts, bile salts, obesity, inflammatory bowel disease, tobacco smoking, and poisons [2]. Our patient did not have any of these risk factors. The most common site of metastasis is the peritoneum, seen 10-20% time at the time of diagnosis. Peritoneal metastases represent an advanced stage of CCA, where the tumor is deemed unresectable, and chemotherapy is initiated.

Based on a clinical trial by Valle et al., a combination of gemcitabine and cisplatin has been established as the gold standard for advanced or metastatic biliary tract cancer [3]. The trial showed median overall survival of 11.7 months in the gemcitabine-cisplatin (gem-cis) group compared to 8.1 months in the gemcitabine-only group. However, gemcitabine therapy has been shown to have an association with hemolytic uremic syndrome.

## Case presentation

**Figure 1:**
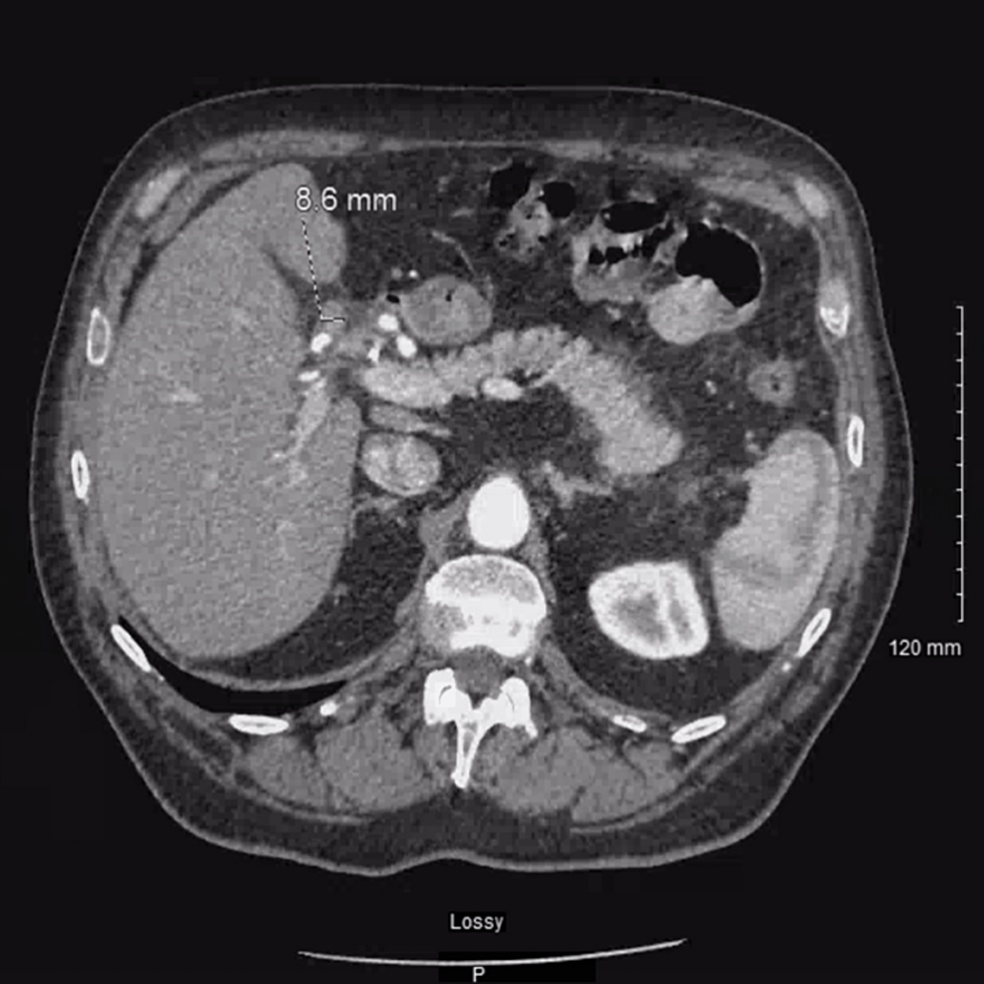
Infiltrative lesion in the porta hepatis which likely represents a Klatskin tumor (labeled)

**Figure 2:**
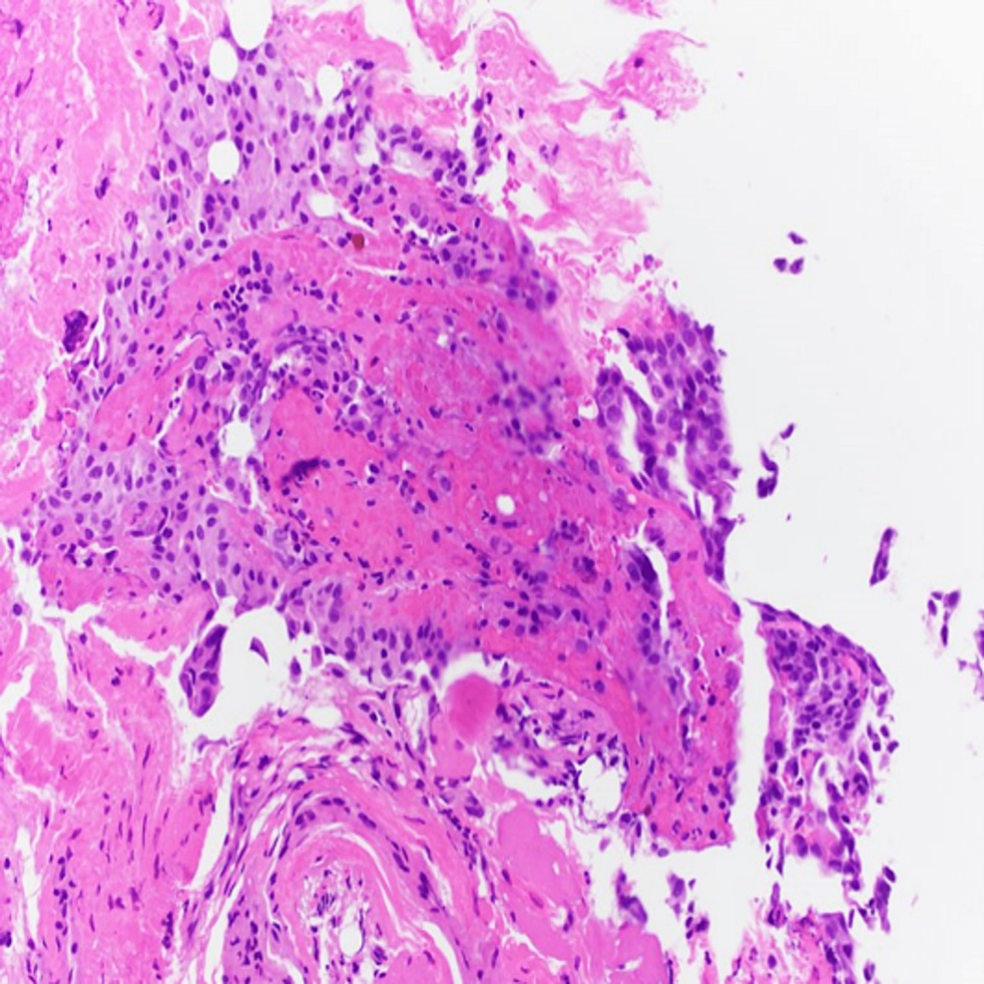
Peritoneal biopsy showing invasive adenocarcinoma, moderately differentiated, pancreaticobiliary type

A 60-year-old male with a history of gastroesophageal reflux disease, malignant melanoma of the skin, heavy alcohol use, and a family history significant for pancreatic cancer in his brother initially presented with diffuse abdominal pain and jaundice. Initial magnetic resonance cholangiopancreatography showed suspected choledocholithiasis. Endoscopic retrograde cholangiopancreatography showed a malignant stricture at the common hepatic duct extending into the hilum. Esophagogastroduodenoscopy was done, and pathology from the extrahepatic bile duct showed atypical glandular cells. Diagnostic laparoscopy showed peritoneal nodules over the right hemidiaphragm and liver surface nodules. Surgical resection was canceled upon seeing metastases. Pathology confirmed adenocarcinoma. He was diagnosed with stage IV cholangiocarcinoma and Klatskin tumor metastatic to the liver and peritoneum. Gem-cis therapy was initiated. Cisplatin was later discontinued due to peripheral neuropathy, and he continued gemcitabine only at 1000 mg/m^2^ every other week. Figure 1 reveals the infiltrative lesion in the porta hepatis representing a Klatskin tumor. Figure 2 reveals histopathological imaging of the peritoneal biopsy showing invasive adenocarcinoma.

 

Fifteen months and nineteen cycles later, the patient was seen for bilateral lower extremity swelling, increased fatigue, and shortness of breath with exertion. Vital signs revealed a blood pressure of 160/86 millimeters of mercury. On physical exam, he had 3+ bilateral lower extremity pitting edema. A transthoracic echocardiogram revealed a preserved ejection fraction of 65%. The iron panel, B12, and folate levels were all within normal limits. Additional tests including antinuclear antibody, hepatitis panel, human immunodeficiency virus, and coronavirus screening were negative. Complement levels C3 and C4 were normal. ADAMTS13 activity was 61%. Gemcitabine was discontinued, with no improvement in symptoms in two weeks. Baseline laboratory investigations before gemcitabine therapy, after the last session of gemcitabine therapy, and two weeks after eculizumab therapy are shown in Table 1.

**Table 1:** Laboratory investigations MCV: Mean corpuscular volume; BUN: blood urea nitrogen; AST: aspartate aminotransferase; ALT: alanine transaminase; ALP: alkaline phosphatase; LDH: lactate dehydrogenase

Laboratory investigations	Results			Reference range
Complete blood count	Before gemcitabine therapy	After gemcitabine therapy	After eculizumab therapy	
White blood cells	4.6x10^9^/L	3.3x10^9^/L	6.3x10^9^/L	4.0-10.5x10^9^/L
Hemoglobin	10.5 g/dL	7.3 g/dL	12.1 g/dL	12.0-16.0 g/dL
Hematocrit	31.2%	21.0%	35.5%	37.0-47.0%
MCV	103.8 fL	112.3 fL	95.9 fL	80.0-100.0 fL
Platelet count	136x10^9^/L	37x10^9^/L	86x10^9^/L	140x10^9^-400x10^9^/L
Comprehensive metabolic panel				
Sodium level	136 mmol/L	130 mmol/L	140 mmol/L	133-147 mmol/L
Potassium level	4.9 mmol/L	3.9 mmol/L	4.5 mmol/L	3.5-5.6 mmol/L
Chloride	101 mmol/L	102 mmol/L	111 mmol/L	96-110 mmol/L
Carbon dioxide	27.0 mmol/L	21.0 mmol/L	26.0 mmol/L	20-32 mmol/L
BUN	18 mg/dL	30 mg/dL	30 mg/dL	5-27 mg/dL
Creatinine	0.74 mg/dL	1.81 mg/dL	1.17 mg/dL	0.40-1.40 mg/dL
AST	92 IU/L	90 IU/L	33 IU/L	15-46 IU/L
ALT	40 IU/L	37 IU/L	20 IU/L	<=49 IU/L
ALP	83 IU/L	91 IU/L	102 IU/L	38-126 IU/L
Total bilirubin	0.5 mg/dL	0.9 mg/dL	0.4 mg/dL	0.2-1.3 mg/dL
Other laboratory values				
Haptoglobin	Not available	<10 mg/dL	118 mg/dL	32-363 mg/dL
LDH	Not available	1302 IU/L	473 IU/L	313-618 IU/L

The diagnosis of hemolytic uremic syndrome was made clinically, with evidence of anemia, thrombocytopenia, and acute kidney injury seen in laboratory values. He was admitted to the hospital and received two intravenous doses of eculizumab 900 milligrams, improving his edema and shortness of breath. Eculizumab was discontinued, and he was discharged in stable condition. 

## Discussion

Hemolytic uremic syndrome (HUS) is a thrombotic microangiopathy (TMA) characterized by the triad of renal failure, microangiopathic hemolytic anemia, and thrombocytopenia [4]. Typical HUS usually follows a gastrointestinal infection, caused by the Shiga toxin produced by Escherichia Coli. Atypical HUS (aHUS) is caused by alternative complement pathway dysregulation and secondary causes including malignancy and chemotherapy [5]. Typical agents reported have been mitomycin, cisplatin, and most recently gemcitabine. Gemcitabine-induced HUS (GiHUS) has a low incidence rate of 0.015% to 0.31% with a high mortality rate as high as 50%, necessitating prompt diagnosis [6]. The diagnosis of aHUS is challenging, and it is made clinically based on the presence of the triad as mentioned while excluding important differential diagnoses, namely thrombotic thrombocytopenic purpura (TTP). Screening for complement protein mutations and antibodies may be performed in aid of diagnosis. However, laboratories that offer genetic testing are not widely available, and it is often not pursued if clinical evidence supports aHUS [7]. In addition, the use of complement levels for diagnosing aHUS is not adequately sensitive [8]. A kidney biopsy may be performed if there is still diagnostic uncertainty. However, kidney biopsy in aHUS is often indistinguishable from other forms of TMA, showing thrombi in glomerular capillaries, mesangiolysis, and endothelial injury [9]. Our patient had a suspected inciting factor and laboratory abnormalities supporting the clinical triad, leading to the diagnosis of aHUS, and thus kidney biopsy was not performed.

The mechanism behind aHUS has been hypothesized to be either the direct spread of cancer or endothelial damage caused by the medications [10]. Studies show that drug-induced HUS almost always presents with severe kidney injury, as seen in our patient. In contrast, drug-induced TTP presents with minimal kidney injury [11]. The most important differential diagnosis was TTP, a common phenotype of TMA, which was ruled out in our patient due to ADAMTS13 activity of 61%. Severe ADAMTS13 activity deficiency is defined as ADAMTS13 <10%, the biological marker specific for TTP [12].

No known mechanism explains why gemcitabine causes HUS. Gemcitabine has no structural similarities to mitomycin, cisplatin, bleomycin, and 5-FU, all of which have been shown to cause HUS [13]. The mean duration of time between the initiation of therapy and the onset of HUS is 7.4 months [14]. In our patient, thrombocytopenia was first noted three months into his treatment with a platelet count of 112 x 109/L, with the onset of worsening symptoms at fifteen months. Studies do not show a strong dose dependence, and HUS can still occur at a lower dose of gemcitabine in combination with other agents, as seen in our case [15].

Traditionally, plasmapheresis has been used in chemotherapy-induced TMA despite literature showing up to 50% of patients progressing to end-stage renal disease. Plasmapheresis also does not play a role in complement-mediated end-organ damage that can be caused by HUS [16]. While plasmapheresis can transiently improve platelet count and lactate dehydrogenase level in some cases, the underlying complement dysregulation and microangiopathic processes are likely to persist. Clinical trials have shown that early intervention with complement inhibitors such as eculizumab can significantly improve renal function and quality of life without serious infection-related adverse events [17]. Eculizumab is a monoclonal antibody directed against C5, blocking the assembly of C5b-C9 complex to prevent endothelial injury. Treatment duration and dosages are debated in most guidelines, with susceptibility of encapsulated bacteria such as N.meningitidis cited as the potential serious adverse event [18]. Our patient tolerated the therapy well without any side effects. In addition, he received both meningococcal and haemophilus influenza type B (Hib) vaccinations during his hospitalization. Research on the feasibility of discontinuing long-term eculizumab therapy is undergoing. A prospective study by Fakhouri et al. showed that in patients with aHUS and no detected rare complement gene variants or complex rearrangements, it was safe to discontinue eculizumab [19]. Female sex and rare gene variants were associated with an increased risk of aHUS relapse. Furthermore, two institutional series reported safe discontinuation of eculizumab among non-dialysis-dependent patients who were in hematologic remission and had a stable renal function. Relapses occurred in 23% of the patients, mostly within three months but all were successfully retreated with eculizumab. Monthly monitoring is recommended for those on stable eculizumab therapy, but more frequent monitoring is recommended for those who discontinued therapy [20]. Given our patient’s rapid improvement, eculizumab was discontinued at discharge, with plans for monitoring laboratory parameters every two weeks at the leukemia clinic and future plans for genetic testing. 

## Conclusions

We present a case of a patient with metastatic cholangiocarcinoma treated on chronic gemcitabine therapy, developing hemolytic uremic syndrome requiring hospitalization. The mainstay management decision is the prompt discontinuation of gemcitabine therapy, and while this is effective in some patients, complement inhibitors may be required for further management of this rare disorder. Eculizumab was well tolerated by our patient. However, given limited research on dosing and the long-term effects of this therapy, he will continue to be monitored very closely.
